# A Quality Control Methodology for Heterogeneous Vehicular Data Streams

**DOI:** 10.3390/s22041550

**Published:** 2022-02-18

**Authors:** Konstantina Remoundou, Theodoros Alexakis, Nikolaos Peppes, Konstantinos Demestichas, Evgenia Adamopoulou

**Affiliations:** Institute of Communication and Computer Systems, National Technical University of Athens, 15773 Athens, Greece; kremoundou@cn.ntua.gr (K.R.); talexakis@cn.ntua.gr (T.A.); npeppes@cn.ntua.gr (N.P.); cdemest@cn.ntua.gr (K.D.)

**Keywords:** data quality control, big data, vehicle sensors, data streams

## Abstract

The rapid evolution of sensors and communication technologies has led to the production and transfer of mass data streams from vehicles either inside their electronic units or to the outside world using the internet infrastructure. The “outside world”, in most cases, consists of third-party applications, such as fleet or traffic management control centers, which utilize vehicular data for reporting and monitoring functionalities. Such applications, in most cases, in order to facilitate their needs, require the exchange and processing of vast amounts of data which can be handled by the so-called Big Data technologies. The purpose of this study is to present a hybrid platform suitable for data collection, storing and analysis enhanced with quality control actions. In particular, the collected data contain various formats originating from different vehicle sensors and are stored in the aforementioned platform in a continuous way. The stored data in this platform must be checked in order to determine and validate them in terms of quality. To do so, certain actions, such as missing values checks, format checks, range checks, etc., must be carried out. The results of the quality control functions are presented herein, and useful conclusions are drawn in order to avoid possible data quality problems which may occur in further analysis and use of the data, e.g., for training of artificial intelligence models.

## 1. Introduction

Due to the enormous amount of data and information that can be collected nowadays through many different sources, it is of great importance to have a complete and self-sustainable system that can measure and evaluate the accuracy, the consistency and, in more general terms, the quality of the data. Especially when the data are collected through sensors, it is useful to have a system that can evaluate the incoming data in almost real time and also provide alerts when something is out of the expected result range.

Additionally, the rapid evolution of Big Data technologies and unlimited scalability options has led to new needs and standards in various domains engaging sensor data. Big Data analytics contain a wide variety of techniques and technologies such as data mining, machine- and deep-learning models and data fusion. The incorporation of Big Data infrastructure in many different domains can lead to remarkable results, but this is highly dependent on the quality of data gathered and analyzed [1].

Accuracy, consistency and relevancy are very important qualities when analyzing streams of data. As long as sensor data arrive with no strict limitations of the predefined protocols or physical limits, the interpretation of the analysis results may be dubious and inconsistent. Moreover, analyzing confounded or contaminated data produces misleading or biased results which are inherently non-reproducible and non-replicable. Therefore, data quality control is not only a useful procedure but, rather, a necessary one that can ensure that certain characteristics are confirmed in the datasets. Especially when the data need to be analyzed in real-time or near-real-time applications, the quality control procedures are very essential for reviewing and creating clear and high-quality data ready to be analyzed and also providing relevant results of high quality. Quality control (QC) usually involves automated and non-automated processes that compare the data to pre-defined standards and requirements. More specifically, in the automated approach, quality control is used for the detection of missing information, errors during the recording/reading of the data and potential outliers that should be flagged or not in order to be taken into account in the analysis. The comparisons can be made either by independent and point-to-point evaluations, where single data points are checked and compared to the defined standards or to other data in order to find the inconsistencies, or even through trend analysis where statistical trends and patterns are identified using pre-existing benchmarking or historical data [2].

In this study, a concise presentation of the infrastructure of a hybrid Big Data platform, which receives data streams from vehicles, is provided. The collection of these data was the initial step needed to serve the purpose of this study, which examines the quality control actions that can be taken for this specific dataset in order to evaluate the quality of the data. The data collected have various formats which impose the necessity of using different QC actions based on the format and nature of them. 

The remainder of the paper is structured as follows: Section 2 contains a brief literature review on quality controls. Section 3 presents the hybrid Big Data platform used for data collection and hosting, as well as the data format used. Section 4 gives an overview of the proposed quality control actions examined in the context of this study whilst Section 5 presents the analysis results produced, and Section 6 features a general discussion and concludes the study.

## 2. Related Works

Sensor data are used in many applications in everyday life. As mentioned in [3], data obtained through sensors are mostly susceptible to errors such as outliers or anomalies. Outliers actually declare whether the data do not belong to the dataset distribution as was defined by the previous data (pattern) or exceed the defined thresholds [4]. In addition, missing data are a very common issue that occurs in sensor data which can be caused by network errors, connection malfunctions, device battery life being low and many other reasons [5]. Teh et al. [3] also mentioned bias and stuck-at-zeros as common faults when handling sensor data; in the first case, “a value that is shifted in comparison with the normal behavior of a sensor” and, in the second case, “values that are constantly at zero over an extended period of time”.

In [6], Taylor and Loescher described a three-stage approach to deal with this kind of fault, including tests, such as plausibility and threshold tests, as well as some of distribution tests using the standard deviation of the data, to check for fluctuations and outliers. In their analysis, they used statistical tests, such as the sigma test, delta test, step test, null and gap test, in order to determine that the data followed a predefined pattern and were not missing. They also used the Gaussian distribution to define the threshold and the limits of their testing. On the other hand, Ye et al. [7] proposed a distance-based approach in order to detect anomalies or faults in the sensor readings. As Ye et al. [7] mentioned in their research, addressing the anomalies of the sensors is more difficult than just addressing issues such as values falling out of range or missing. Thus, they proposed the CBLOF method, using distances to determine the statistically important fluctuations between the readings. Moreno-Tejera et al., in [8], explained the importance of the correct timestamps in the results of the quality control procedures and the overall analysis, as well as other techniques of quality control. Following the same direction, Liu et al. [9] proposed a chance-constrained programming model that determined the optimal strategy for allocating the control resources to mitigate the data quality problems of a data warehouse by engaging a modified artificial bee colony algorithm.

## 3. Platform Design and Data

### 3.1. Hybrid Big Data Platform

As mentioned by McAfee and Brynjolfsson, Big Data systems should address the three vs. of data namely: volume, velocity and variety [10]. The data studied for the purposes of this study were compatible to the definition given by McAfee and Brynjolfsson and were hosted in a hybrid Big Data platform. The data stored in this platform were streamed from and to vehicles in near real time using Big Data technologies.

The platform was developed mainly to host vehicular data in a modular way and consisted of multiple components which interacted with each other and provided an integrated solution. The main technologies and software components integrated in this platform were:

- Flask. It is a Pythonic microweb framework which renders the process of web application design and deployment much easier by incorporating the client–server architecture which is essential for such applications as it enables data transfer between the client(s) and the server [11].
- Hybrid database system. This component consisted of a NoSQL database, in this case, the MongoDB, which covers the Big Data storage needs, as well as a SQL database which aids the real-time needs by retrieving, streaming and storing small amounts of data.
- Communication interfaces. The communication between the platform and the vehicles was achieved using REST APIs which are offered by the Flask microweb framework as it is described above. This development choice enabled two-way communication from and to the platform, as well as from and to the vehicles and external applications [12].
- Big Data framework. The NoSQL database, as well as the Big Data needs, was covered by the Hadoop framework [13] engaged in the platform. Hadoop facilitated the connection requirements with the NoSQL database, as well as the Big Data operations needed.
- Data analysis and reporting. Alongside the Hadoop framework, the platform integrated a data analysis and reporting component which engaged artificial intelligence (AI) services in order to provide timely analyses of the data based on pre-trained models so as to produce periodical reports and near-real-time alerts/messages to vehicles or external applications.

**Figure 1:**
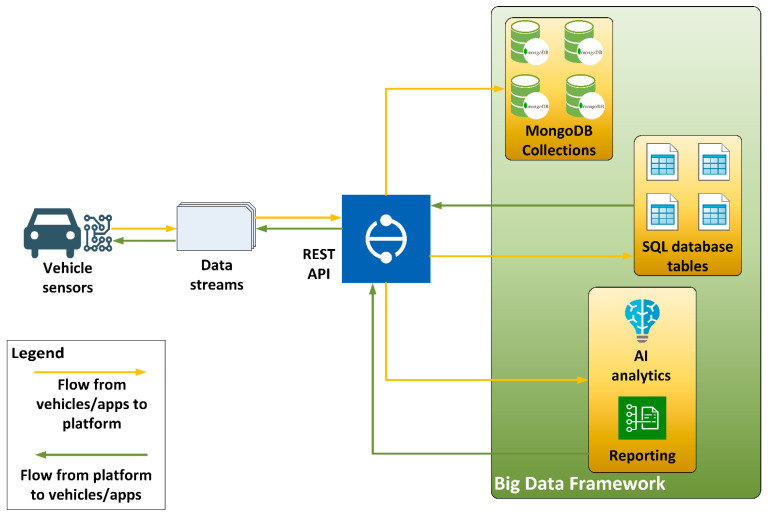
Data flow between vehicles and the hybrid Big Data platform.

A schematic overview of the conceptual data flow between vehicles and of this very platform are depicted in Figure 1.

The design and the development of such solutions does not come without costs or risks. The increased analytics demands require advanced machine-learning models and procedures which, in their turn, demand high computational capabilities and, thus, expensive hardware. The need for high availability imposes the need for high-end software and to a redundancy of hardware resources which lead to higher acquisition and operational costs. Another risk that should be taken into account when considering data analysis, which is directly connected to the purpose of this study, is data quality control, as, in many cases the streaming of data fails because of faulty streaming equipment or due to network problems, thus, potentially leading to wrong analysis outcomes and predictions. In addition to this, and due to the always-online nature of the platform, disconnections or network unavailability could also lead to undesirable behavior. Given the aforementioned risks, which also may affect the data quality and availability, it was of high importance to engage data quality control procedures, as described later in this study.

Regarding security measures taken to avoid breach phenomena, non-repudiation was achieved through cryptography (e.g., digital signatures) and included additional services for authentication, auditing and logging. Moreover, the integration and the demand for credentials by the provided REST API routes ensured that a part of the suggested platform could not deny the authenticity of the data sent from the car’s sensors, a measurement which offered non-repudiation. Other security breach mitigation measurements adopted in the platform included the use of SSL certification and the configuration of the machines hosting the platform firewall which provided limited access to specific cars for each distinct route, as well as a scheduled procedure for a monthly kernel update on the machines where the APIs were hosted.

### 3.2. Collected Data

The data analyzed for this system were collected from an on-board diagnostics (OBD) unit attached and suitably connected to a vehicle. The data were sent to the database in almost real time every few seconds while the engine was running.

The data that were collected contained 19 different variables/measurements with different formats. The number of observations was 597,549 instances. The variables, along with their descriptions, can be found in Table 1:

Based on the analysis of the above data, the decision was made to separate the variables into four categories based on their format and nature. Those four categories were namely:

- the character/string variables;
- the numerical variables;
- the categorical variables; and
- timestamp.

This separation helped to define a specific approach to the quality control actions for each of the categories mentioned above and allowed us to have control of the data.

More specifically, the first category of character/string data included the ID, the carPlate, pendingTrouble and vinNumber variables, while the second and the biggest category of the numerical data included SpeedOBD, accuracy, altitude, bearing, engineRunTime, engineTemp, fuelLevel, intakeTemp, relThrottle, rpm and speedGPS. Regarding the third category—the one including the categorical data—this consisted of the fuelType and, finally, the last category included the timestamp.

## 4. Proposed Quality Control Actions

The overall scope of the variables’ categorization into four categories, as presented in Section 3.2., was to apply different types of quality control procedures to each category based on their specific nature and reason for collection. Therefore, a detailed presentation and explanation is presented below featuring the different types of controls for each category. However, certain techniques were applied to all the categories, such as missing value checking and format checking, to ensure that our data followed the predefined format, as well as that there were not any missing data that would render the rest of the analysis problematic.

Following the missing value checking—which was basically a simple binary check of whether there was an output of the sensor or not—applied to the entire dataset, the format checking [14], as already mentioned, ensured that the data followed a predefined pattern. For each category of variable, a different check was performed according to the nature of the data. For instance, for the string variables, a sequence of characters or a mixture of characters, symbols and numbers was expected for each input, e.g., for the variable carPlate, the expected format was XXX-1234 (three capital letters, one dash symbol and four numbers). Knowing the format of the variable beforehand made this checking feasible. The same principle was applied for the timestamp variable, which must fit the DDMMYY_HHMMSS format. On the other hand, the numerical variables—those of categories 2 and 3—were checked according to their numerical values. This could also have been performed by checking if the specific variables were numeric or integers.

After the missing data and format matching checks were performed, the next steps could be carried out by engaging different techniques and control methods to each of the categories. Starting from category 1 and the string/character variables, format checking and missing values checks were deemed adequate in order to ensure the desired quality of the data. On the other hand, for the second category of the numerical data, further control procedures needed to be applied, such as:

- range check;
- outlier check; and
- sudden fluctuation check.

For the third category of the categorical data, in addition to the missing data check and the format check, a range check also needed to be performed. Last but not least, for the fourth category, including the timestamp data, the following checks were performed:

- duplication check; and
- sequential check.

A range check ensures that all values fall within established upper and lower bounds. Bounds can be established based on the specific sensor limitations or can be based on historical, seasonal or finer timescale ranges determined for each data variable or measured attribute. On the other hand, a fluctuation test checks for sharp increases or decreases from the expected value in a short time interval, such as a spike or step function. These tests often employ statistical measures, such as the standard deviation of the preceding values, for detecting outliers or spikes that deviate by more than 2 or 3 sigma (standard deviations) from what is expected. An alternative solution is to check if the median value of points t, t + 1 and t − 1 does not exceed a fixed magnitude from point t. The duplication check ensures that there is only a unique value in a short—or large—time interval of data, which is a major factor when handling timestamps, including date and time, whereas the sequential check ensures that the next value collected is timely next to the previous one [15,16].

Outlier checks are indicative of the pattern of the variables and the correlation of one to another. In general, outliers can be defined as data points that are significantly distanced from the main volume of the collected data [17]. Outlier values, in most cases, indicate an abnormal behavior or an anomaly in the examined data. Furthermore, these data points often refer to data or measurements which are possibly met on rare occasions [18]. These checks are necessary to ensure that good data quality is maintained in continuous collection. For instance, in the dataset we have already presented, it is obvious that the values of the speed of the OBD and the speed of GPS should be equal or have a small difference, as well as have the same direction, meaning that, when one increases, the other one should also increase and vice versa. All of those techniques can also indicate a potential accident, since, when an accident occurs, the sensors would most probably provide strange or corrupted data; however, this is a study that it is not covered in this paper.

Most of the above techniques can be implemented, especially if the range of the data is known or batches of historical data are available, and can indicate the pattern, the bounds and the correlation of the variables. In Table 2, there is a summarization of the proposed actions and related works that are relevant.

## 5. Analysis on Benchmark Data and Testing for Quality

Based on the proposed data quality actions presented in Section 4, this section further features the analysis of the available data carried out to determine some of their main quality measures and characteristics. This analysis was mostly based on the numerical data of the second (the numerical variables) and third category (the categorical variables), since the first (character/string variables) and fourth category (timestamp) included data that were characters and/or specific-format-based. More specifically, we examined 597,549 observations with no missing values. Table 3 summarizes the statistics of the available data.

The above statistics offered useful insights for determining the range of the measurements which helped assess the limits of the variables so as to appropriately flag every observation that arrived. Moreover, the mean of the variables could be determined and, thus, by combining that value with the standard deviation, every observation could be flagged as an outlier or not—taking into account whether that variable followed a normal distribution. This was approximately true due to the size of the sample chosen each time for measuring the above statistics. More precisely, the empirical rule [19]—or three-sigma rule—gave us the upper and lower control limits in statistical quality control by assessing every value that fell out of the three-deviations rule as an outlier or error. This analysis could be repeated for the data received through time in order to update the distribution of the data and achieve more representative results. This approach updated the mean and standard deviation of the variables so as to determine new upper and lower limits.

**Figure 2:**
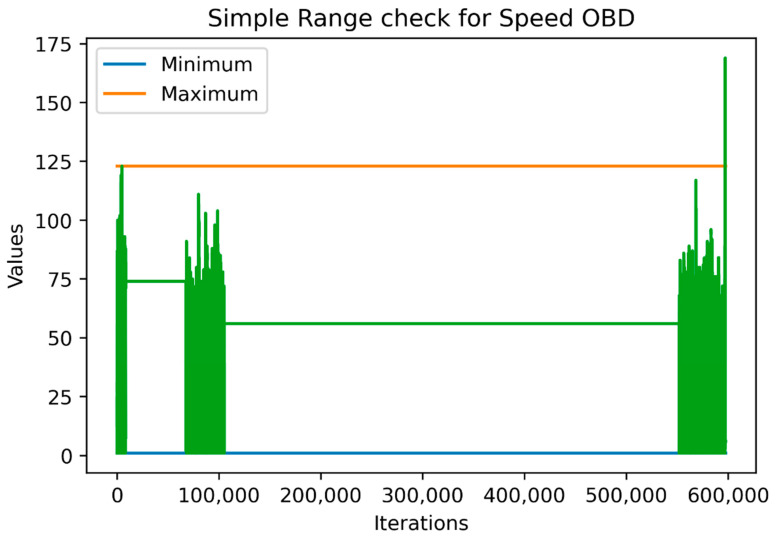
Simple range check based on the historical data.

**Figure 3:**
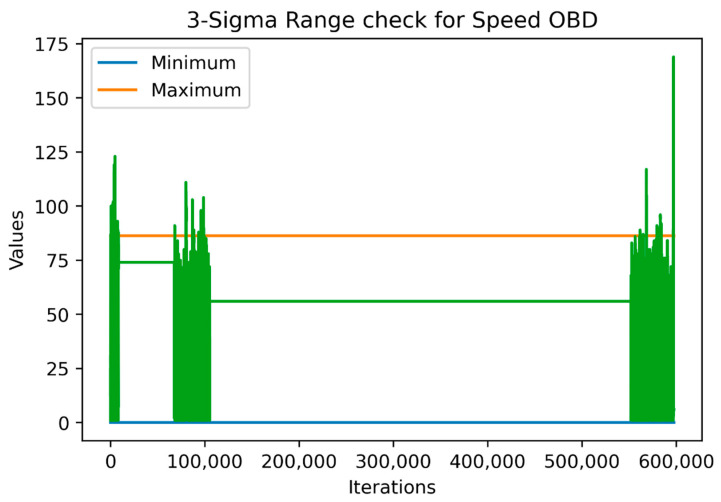
Range check using the “three-sigma” approach based on the historical data.

Thus, for numerical variables, the data were separated into training validation and testing datasets, a procedure that was similar to machine-learning models training. Figure 2 and Figure 3 show the range and outlier checks based on the simple range and three-sigma approaches carried out, respectively, on the training data (the last 1000 values being the testing data). For the sake of simplicity, we selected the SpeedOBD variable for the figures.

As shown in the above figures, the “three-sigma” approach is stricter than the simple approach, which means that it might include some outliers that were not actually out of the ‘logical’ ranges the variable were expected to take. On the other hand, since the simple approach is greedier, it was affected by the outliers and fluctuations observed in the historical data. For example, if we had included the whole sample as a training sample for the simple approach, we would have achieved a maximum value equal to the larger green vertical line reaching almost 175 km/hour which may be feasible but is definitely an outlier and may need to be checked. Lastly, both approaches agreed that the minimum value could not be below zero.

Table 4 features all the characteristics mentioned above in order to identify the potential outliers by performing a range and outlier check through the simple range and three-sigma approach. The Test Data columns indicate the number values found in the test dataset that were outside the ranges indicated by the two approaches.

As we can see, the results we achieved for both approaches were similar for some variables whilst the SpeedOBD, accuracy, intakeTemp and relThrottle variables differed. Looking back on Table 3 and the data statistical measures, we can see that, for the SpeedOBD variable, the mean was 58.010 with a standard deviation of 9.529, and this is the reason why the three-sigma approach range was narrower. Regarding the accuracy of the results, the range was significantly reduced by the three-sigma approach, and this happened due to the low mean found earlier in Table 3. For the intakeTemp variable, there was a mean of 25.87 with a standard deviation of 3.8, which means that the values were mostly concentrated around and very close to the mean, thus, making a seemingly narrower range for the three-sigma approach. For the relThrottle variable, the three-sigma range was, again, smaller than the simple range approach as the mean value of this variable was 99.270 and the standard deviation 6.476 which means that the values belonged to a narrow range around the mean value.

In Table 4, since the minimum acceptable value for all variables was 0 due to the logical expectation when the vehicle is not moving, we tested the values on that minimum and not the one indicated by the “three-sigma” approach to find the outlier values. Thus, it is worth mentioning that the outliers’ detection analysis had similar results for most cases expect for the intakeTemp variable. This happened due to the fact that the values of this variable were very close to the mean value compared to every other variable examined.

## 6. Discussion and Conclusions

The quality control procedures and analysis presented above indicated that, in large datasets where data are acquired rapidly, these methods reveal hidden patterns and useful insights which can lead to enhanced reasoning. The QC in vast data streams proves to be a necessary analysis as it indicates possible problems that are directly correlated with the data and could possibly lead to problems such as the incorrect training of machine-learning models or the drawing of wrong conclusions. As the amount and the complexity of data increase rapidly from day to day in various domains, including vehicular and traffic data, the quality tends to decrease; thus, it is of utmost importance to take QC actions to enhance their quality. Although such methodologies and actions can offer useful insights about the data attributes, the quality evaluation, as well as the appropriate analysis methods applied, should always be conducted by experts who can evaluate the analysis results and draw useful conclusions.

In addition to the QC actions mentioned in this study, a distance-based algorithm would be very beneficial for the determination of the estimation and the flagging of potential fluctuations of the continuous measurements received by the sensors. Such an algorithm could be the KNN, as described in [20], in order to assess whether the upcoming values fall inside the expected pattern or not. Another future research direction is to explore further the capabilities of the methodology presented in this study in another dataset: either a larger one with the same data or a totally different dataset. Moreover, the usage of data after the quality control actions and the proper preprocessing applied can be evaluated compared to unprocessed data without any quality control actions to benchmark the results and predictions of different artificial intelligence (AI) models and algorithms.

## Figures and Tables

**Table 1:** Description of variables.

Variable	Description
@_id	a unique ID string variable corresponding to each of the observed measurements given by the OBD
SpeedOBD	a numerical variable in km/h of the speed measured by the OBD attached to the vehicle
carPlate	a string variable of the car plate
accuracy	a numerical variable of the geolocation accuracy of the vehicle
altitude	a numerical variable of the altitude from sea level measured in meters based on the vehicle’s geolocation
bearing	a numerical variable measured in degrees of the vehicle’s direction
engineRunTime	a numerical variable of the total time in hours which the engine of the vehicle was actively running
engineTemp	A numerical variable of the current temperature of the engine at the time the measurements were recorded, in Celsius
fuelLevel	a numerical variable of the percentage of the fuel the tank of the vehicle had at the moment the measurement was recorded
fuelType	a numerical categorical variable indicating the type of fuel the vehicle uses
intakeTemp	a numerical variable of the environmental temperature measured in degrees Celsius
lat	the latitude of the vehicle location at the time the specific measurement was recorded
lon	the longitude of the vehicle location at the time the specific measurement was recorded
pendingTrouble	a string variable indicating whether there was any alarming event or problem based on the diagnostics given by the OBD unit on the vehicle
relThrottle	a numerical variable of the percentage of the throttle’s position the moment the measurement was taken
rpm	a numerical variable for the vehicle’s engine revolutions per minute
speedGPS	a numerical variable in km/h of the speed measured by the GPS of the smartphone connected via Bluetooth to the OBD unit of each vehicle
timeStamp	the date and time the measurement was taken in the format DDMMYY_HHMMSS
vinNumber	a string variable indicating the unique vehicle identifier given by the manufacturer

**Table 2:** Proposed quality control actions and related works.

Action	References
Missing data	[5,6]
Format check	[15]
Range check	[6,15]
Outlier check	[6,7]
Sudden fluctuation check	[7]
Duplication check	[8]
Sequential check	[8]

**Table 3:** Data statistics values.

Variable	Samples	Minimum	Maximum	Mean	Std. Deviation	Variance
SpeedOBD	597,549	1	169	58.010	9.529	90.797
accuracy	597,549	3.802	3099.999	17.983	100.158	10,031.703
altitude	597,549	0	402	208.910	69.269	4798.149
bearing	597,549	0	359.990	206.053	103.631	10,739.424
engineTemp	597,549	−40	102	65.850	15.656	245.106
fuelLevel	597,549	0	100	90.810	19.345	374.238
intakeTemp	597,549	0	64	25.870	3.862	14.916
relThrottle	597,549	0	100	99.270	6.476	41.936
rpm	597,549	0	10,159	1980.360	281.645	79,323.843
speedGPS	597,549	0	142	40.490	33.197	1102.044

**Table 4:** Results of simple and three-sigma approach, as well as outliers detected in the numerical data.

	Train Data				Test Data	
	‘Simple Range’ Approach		‘Three-Sigma’ Approach		Number of Outliers Found by the ‘Simple Range’ Approach	Number of Outliers Found by the ‘Three-Sigma’ Approach
	Minimum	Maximum	Minimum	Maximum		
SpeedOBD	1	123	28.78	86.28	3	8
accuracy	3.802	3099.99	−282.73	318.70	0	0
altitude	0	402	1.44	416.59	0	0
bearing	0	359.99	−104.79	516.95	0	0
engineTemp	0	102	19.65	112.12	386	386
fuelLevel	0	100	33.34	148.44	0	0
intakeTemp	0	64	14.59	37.26	0	164
relThrottle	0	100	80.16	118.42	0	0
rpm	0	4609	1407.77	2542.97	389	392
speedGPS	0	142	−59.08	140.07	0	0

## Data Availability

The data presented in this study are available on request from the corresponding author.
